# Non-Tuberculous Mycobacterial Diseases in Children

**DOI:** 10.3390/pathogens9070553

**Published:** 2020-07-09

**Authors:** Aniello Meoli, Michela Deolmi, Rosanna Iannarella, Susanna Esposito

**Affiliations:** Pediatric Clinic, Pietro Barilla Children’s Hospital, Department of Medicine and Surgery, University of Parma, via Gramsci 14, 43126 Parma, Italy; Aniello.meoli@gmail.com (A.M.); michela.deolmi@studenti.unipr.it (M.D.); rosanna.iannarella@hotmail.it (R.I.)

**Keywords:** cystic fibrosis, lymphadenitis, *Mycobacterium tuberculosis*, non-tuberculous mycobacteria, non-tuberculous mycobacterial disease

## Abstract

Non-tuberculous mycobacteria (NTMs) are ubiquitous and opportunistic emerging bacteria with the potential to colonize and eventually infect either immunocompromised or immunocompetent individuals. In the last three decades, the prevalence of disease caused by NTMs has increased in several countries. The increased prevalence of NTM infection can be explained by an ageing population with rising comorbidities, HIV infection, the common use of immunosuppressive drugs, and improved diagnostic methods. The aim of this review is to demonstrate the clinical relevance of NTMs in children, describing their features and manifestations, diagnostic tools, and therapeutic approaches. We collected data from the literature about NTM infections in young patients over the past five years (2014–2019) using the keywords “non-tuberculous”, “mycobacteria”, “paediatric”, “NTM”, “cystic fibrosis”, and “children”. Recent literature points out that NTMs are ubiquitous, with several species including both those that are pathogens for humans and those that are not. This means that, if a mycobacterium is isolated from a patient’s specimen, we have to distinguish between a simple colonization and an NTM-related disease. The start of treatment depends on many factors that are necessary to consider, such as clinical and imaging features, patient comorbidity and immunocompetence, drug adverse effects, and compliance with a very long therapy that can last many months. Due to the increasing prevalence and clinical relevance of NTMs, guidelines for their optimal management, especially in the presence of chronic underlying disease, are urgently needed.

## 1. Introduction

Non-tuberculous mycobacteria (NTMs) are ubiquitous and opportunistic emerging bacteria with the potential to colonize and eventually infect either immunocompromised or immunocompetent individuals. In the last three decades, the prevalence of disease caused by NTMs has increased in several countries [1,2]. Moreover, the increased prevalence of NTM infection can be explained by an ageing population with rising comorbidities, HIV infection, the common use of immunosuppressive drugs, and improved diagnostic methods [1]. The aim of this review is to demonstrate the clinical relevance of NTMs in children, describing their features and manifestations, diagnostic tools, and therapeutic approaches. We collected data from the literature about NTM infections in young patients over the past five years (2014–2019) using the keywords “non-tuberculous”, “mycobacteria”, “paediatric”, “NTM”, “cystic fibrosis”, and “children”.

## 2. Epidemiology

In North America, the incidence of pulmonary infections by NTMs is actually higher than the incidence of tuberculosis; clearly, this increase in incidence has implications for the prevalence of multiple NTM infections and the mortality rates associated with these infections. The prevalence of this type of infection in the U.S. increased from 20 to 47/100,000 in the population between 1997 and 2007 with a further build-up by at least another twofold between 2010 and 2014 [2]. Similarly, in different regions of the world, the prevalence of NTM infection has risen: in Australia from 2.2 to 3.3/100,000 in the population between 1999 and 2005 and in Canada from 9.1 to 14.1/100,000 in the population between 1997 and 2003. In Europe, lower numbers are usually reported: in England, Wales, and Northern Ireland, the number of NTM infections increased from 0.9 to 2.9/100,000 in the population between 1995 and 2006, while in Germany, it increased from 2.3 to 3.3/100,000 in the population between 2009 and 2014.

Regarding the paediatric population, international studies have shown an annual incidence of diseases caused by NTMs ranging from 0.8 to 4.5/100,000 in children [1,3]. Different nationwide studies estimated an incidence of NTM infection from 0.6 to 1.6/100,000 children in Australia, 0.77/100,000 children in the Netherlands, and 2.5/100,000 children in Canada [4]. No comparative study is available showing gender differences in susceptibility to NTM infections. 

## 3. Classification and Pathogenesis

The term NTMs includes all mycobacteria that do not belong to the *Mycobacterium tuberculosis* complex and *M. leprae* and encompasses more than 170 species with different capabilities of adaptation to specific environmental conditions and occurrence for each world region [1,5]. Humans have always been surrounded by these opportunistic pathogens, which may inhabit several ecological niches, such as soil, surfaces, water reservoirs, and food and animals, based on analysed species [6].

Two characteristics that make them highly adaptable are their ability to form biofilms and their greater resistance to common water disinfectants in comparison with other microorganisms [7]. The environmental nature of NTMs together with the ability to assemble biofilms on different surfaces and intracellular persistence plays a key role in their pathogenesis. The transmission of NTMs occurs through exposure to contaminated aerosols, dust particles, or water, but the transmission from person to person has not been documented despite some genetic analysis on certain strains of *M. abscessus* in patients with cystic fibrosis revealing the possibility of this type of transmission via fomites and aerosols [1,7]. NTMs can affect any organ in the body, although the main clinical manifestations in adults are pulmonary disease (NTM-PD) and disseminated mycobacteriosis, while in children, local or generalized lymphadenitis is reported, though skin, soft tissue, pulmonary, central nervous system, and disseminated infections are also reported [6]. 

NTMs can be divided into slow-growing mycobacteria (SGMs) and rapid-growing mycobacteria (RGMs), as summarized in Table 1. The first group belongs to the *Mycobacterium avium* complex (MAC, which includes *M. avium* and *M. intracellulare*) and *M. gordonae*, which is the most important and widespread in the environment, while the second group belongs to the *M. abscessus* complex (MAbsC, differentiated into subspecies *M. abscessus*, *M. massiliense,* and *M. bolletii*), which is one of the most frequently isolated *Mycobacterium* species in patients with cystic fibrosis [1,6]. Among the variety of NTM species, differences in pathogenicity have been documented; *M. gordonae* or *M. fortuitum*, which are often found in the environment, have very low pathogenicity, while *M. ulcerans* and *M. marinum* are associated with disease in many cases. Finally, some NTM species can cause defined disease patterns in non-HIV-infected patients, as shown in Table 2 [1].

## 4. Diagnosis

To diagnose NTM disease, the following criteria should be met: (1) clinical and/or imaging features are suggestive of NTM infection; (2) other conditions with similar clinical presentation can be excluded; (3) symptoms do not improve if treatment for other possible diagnoses is provided; (4) laboratory tests detect NTMs in sterile samples (once contamination is excluded) or in non-sterile samples at least two different times [1].

### 4.1. Screening Test

Conventional tuberculosis (TB) screening tests are less useful in NTM disease diagnosis. In particular, the tuberculin skin test shows cross-reaction between TB, BCG immunization, and some NTM species, leading to false-positive results in 30–60% of patients. IGRA (interferon—gamma test) is more specific for TB because it uses 6-kilodilation early secreted antigenic target (ESAT–6) and culture filtrate protein (CFP)–10 peptides, which are not expressed by BCG and by the majority of NTMs, avoiding cross reactions and showing a high rate of true-negative results. Some NTM species, such as *Mycobacterium marinum*, *M. kansasii*, *M. szulgai,* and *M. flavescens*, can cause false-positive results [4].

### 4.2. Laboratory Test

Culture in both liquid and solid media is the gold standard for NTM diagnosis. Nevertheless, it shows a 67.2% sensitivity rate, and NTM growth usually takes 42 days in liquid culture and 56 days in solid culture, leading to a delayed diagnosis and treatment start [1]. Hence, PCR (polymerase chain reaction), which is the most sensitive laboratory tool (92%), regardless of whether a clinical sample is tested, can be used to detect NTM infections more rapidly (i.e., results are available in 12–48 h). Microscopy for acid-fast bacilli has only 35.7% sensitivity and, therefore, has less application in clinical practice [8].

### 4.3. Common Clinical and Laboratory Findings

Non-tuberculosis mycobacteria disease, simulating TB infection, can sometimes present with constitutional symptoms. They include low-grade fever, especially at night, generalized malaise, and rarely weight loss or night sweats. 

Regardless of clinical presentations, laboratory findings show normal or mild elevation of inflammatory parameters such as total white blood cell, neutrophil, and lymphocyte count; C reactive protein; and erythrocyte sedimentation rate [8].

## 5. Medical History: Which Risk Factors?

Medical history is usually negative in children suffering from NTMs [8]. The literature highlights some conditions predisposing NTM colonization that can be divided into systemic and local risk factors, as shown in Table 3. In particular, systemic risk factors include immunocompromised patients such as individuals suffering from severe combined immunodeficiency (SCID), chronic granulomatous disease (CGD), haematological malignancies (in particular chronic lymphoid leukaemia), and graft-versus-host disease (GVHD) or patients affected by HIV with a CD4+ T cell count below 50/µL.

Iatrogenic immunosuppression (i.e., systemic or inhaled steroids; TNF-alpha inhibitors; medications administered after organ transplantation) have also been linked to NTM infections. Moreover, some genetic polymorphisms or mutations causing a reduction in IFN-gamma production or signalling seem to increase the susceptibility to NTM colonization. This is called Mendelian susceptibility to mycobacterial disease and is due to mutations in the *IFNGR1*, *IFNGR2*, *STAT1*, *IL12B*, *IL12RB1*, *TYK2*, *IKBKG*, *CYBB*, *ISG15*, and *IRF8* genes.

Last, vitamin D deficiency or vitamin D receptor polymorphisms seem to be related to NTM infections because they occur mainly during the winter months [9,10].

On the other hand, local risk factors include conditions that cause disruption of the skin barrier and promote NTM colonization (i.e., skin laser cosmetic surgery, LASIK, catheters) or lung disease, such as cystic fibrosis, especially in adolescents, bronchopulmonary aspergillosis or *Staphylococcus aureus* colonization, bronchiectasis itself, tuberculosis, chronic obstructive pulmonary disease (COPD), asthma, and every condition that alters parenchymal structure and bronchial epithelium integrity (i.e., air pollution in industrial areas causes the production of reactive oxygen species and secondary inflammatory responses in the lung) [9,11,12].

## 6. NTM Lymphadenitis

The most common clinical manifestation of NTM disease in immunocompetent children is cervical lymphadenitis [1,13]. The peak incidence is in healthy children between 1 and 5 years of age. The typical entrance site is the oropharyngeal mucosa, as children bring contaminated objects to their mouths; less frequently, the infection occurs through skin lesions [1]. The frequency of individual NTM species is dependent on the geographical location, but the main causative agent is MAC followed by *M. haemophilum*, *M. kansasii*, and *M. scrofulaceum* [13]. 

In population-based surveillance studies, estimates of the annual incidence of NTMs in children range widely from 0.8/100,000 children < 15 years in Australia to 3.1/100,000 children < 15 years or 11.3/100,000 children < 5 years in Germany and 0.8/100,000 children < 18 years in the Netherlands [14]. In children, NTM infection typically affects cervicofacial lymph nodes and manifests itself with an enlarged lymph node in the laterocervical region that appears as a painless lump that can spontaneously drain within weeks/months. Typically, there are no systemic symptoms, such as high fever, lack of response to non-specific antibiotics and a course of disease for more than two weeks (Table 4).

The tuberculin skin test with two PPD tuberculin units is indicative of mycobacterial contact (also following vaccination with BCG) but does not allow differentiation between infection and/or disease caused by *M. tuberculosis* and NTMs. An interferon-γ (IGRA) test is useful because of the greater specificity for *M. tuberculosis* cross reactions with false-positive results in IGRA possible only for *M. kansasii*, *M. marinum*, *M*. *flavescens,* and *M. szulgai*. In the literature, only one-third of patients show an induration greater than 10 mm [15].

Needle aspiration samples (FNA) and/or adenitis biopsies are important for microbiological and histological confirmation since the type of bacterium can be identified by NTM microbiological tests (culture and/or PCR) for MAC. In relation to these two diagnostic methods, Willemse et al. showed that culture and PCR sensitivities were only 41.8% and 71.6%, respectively [15]. A sonographic exam is useful in the pre-surgery phase, while chest X-ray may be part of the differential diagnosis.

### 6.1. Surgical Therapy

Surgery is the gold standard treatment for cervical lymphadenitis. Many authors have shown that surgery is effective, having an 81–100% cure rate with primary excision versus 45% for incision and drainage [1,13,14,16]. Then, the advantage of surgery is to allow histological and microbiological diagnosis.

The main complication of surgery is lesion of the facial nerve, although it is rare: complete excision was associated with a 10% risk of facial nerve palsy, but permanent palsy complicates approximately 2% of the cases; the risk depends on the proximity of the involved lymph node to the facial nerve [13,17]. Early detection and intervention are important to reduce the risk of iatrogenic nerve lesions [17]. Surgical drainage is not considered a valid alternative due to the possibility of complications such as chronic fistulas or scars.

### 6.2. Antibiotic Therapy

Antibiotic therapy is a combination of clarithromycin or azithromycin, rifampicin, and ethambutol (Table 5) and is recommended with respect to monotherapy. However, some authors suggest the use of clarithromycin in cases with a favourable prognosis and without indications for surgery.

Lindeboom et al. had a time to resolution of 9 months with antibiotic therapy in a prospective study, and Gallois et al. had a time of resolution of approximately 6 months [18,19]. No comprehensive data are available on drug–drug interaction and safety of antibiotic therapy for NTM infections and future studies in this regard are needed. 

### 6.3. “Wait-and-See” Therapy

Gallois et al.’s study suggests a possible management for chronic and/or atypical adenitis (more than 3 weeks of evolution without response to antibiotic treatment): surgery, antibiotic (ATB) treatment, and wait-and-see therapy [18,19]. The ideal treatment in the absence of complications (facial nerve injury) is surgery, but these authors suggest antibiotic therapy in fluctuant lesions and wait-and-see therapy in firm lesions through close clinical follow-up; however, “No intervention” was associated with delayed resolution according to Zimmerman et al. (6–12 months) [13].

Furthermore, in the most important studies without intervention, approximately one-third of the cases were caused by *M. haemophilum*, which has a lower pathogenicity than *M. avium*; meta-analysis and the risk of scarring must be assessed.

## 7. NTM-Pulmonary Disease and Cystic Fibrosis 

Cystic fibrosis (CF) is one of the most important local bronchopulmonary risk factors for the development of significant pulmonary disease due to NTMs in children; CF patients have the highest prevalence of NTM-PD compared to other diseases [1,20]. The real prevalence of NTMs in these patients is difficult to estimate, although recent multicentre studies registered a prevalence of 13% in the USA and 6.6% in France [12]. The largest studies prior to 2000 registered a median prevalence of 9%, and those after 2000 registered a median prevalence of 13%, thus highlighting a significant increase in their detection in the last two decades, in the general population [21].

Different studies demonstrated a risk associated with NTM positivity for intravenous antibiotic therapy, corticosteroid treatment, *S. aureus* colonization, and allergic bronchopulmonary aspergillosis (ABPA); more controversy exists regarding the impact of azithromycin treatment on NTM infection, described as a predisposing factor by some authors, a protective factor by others, and as irrelevant by most [12].

NTM species’ prevalence within CF patients is different between the various geographical areas: the most frequent species isolated in North America is the *Mycobacterium avium* complex (MAC), followed by the *M. abscessus* complex (MAbsC), while in Western Europe and Israel, MAbsC is more frequent than MAC [22].

The clinical presentation of pulmonary disease due to MAbsC is usually more severe, and the affected patients are younger at the time of infection and require more frequent intravenous antimicrobial treatment than lung disease due to MAC, which is in accordance with Catherinot et al.’s study [12,21].

The impact of an active MAbsC infection on the lung function of children with CF is, as usual in these patients, measured as an FEV1 reduction that is faster than that observed with other chronic infections such as MAC, *Burkholderia cepacia,* and *Pseudomonas aeruginosa*; precisely, this FEV1 decline stands approximately at 6% per year compared to 1% per year in individuals with colonization only [21].

Regarding the transmission of this multidrug-resistant NTM, a recent population genomic analysis highlights that many MAbsC infections are not acquired from the environment by susceptible individuals but via fomites and potentially via aerosols contemplating the possibility of transmission from person to person [23].

### 7.1. Diagnosis

The detection of these troublesome pathogens in sputum may indicate simple colonization rather than active disease; hence, the diagnosis of NTM-PD can be difficult and should be made on the basis of clinical, radiological, and microbiological criteria with appropriate exclusion of other diagnoses according to recommendations from the American Thoracic Society and Infectious Diseases Society of America (ATS/IDSA), which are the same for patients with and without CF (Table 6) [12,20].

NTM-PD should be suspected in patients with worsening respiratory symptoms (not different from those of other CF-related infections that include recurring or chronic cough with sputum production, haemoptysis, chest pain, dyspnoea in association with or without fever, malaise, fatigue, night sweats, and weight loss) and/or declining pulmonary function tests that are not responsive to antibiotic therapy for typical CF-associated infections and optimized airway clearance [21,24].

Regarding radiological investigations for NTM-PD suspects, the chest radiograph is not useful as opposed to high-resolution CT (HRCT), which can highlight findings such as inflammatory nodules, cavitation, and new tree-in-bud opacities (particularly in areas of mild underlying bronchiectasis) capable of supporting the diagnosis despite being not specific and common in cases of infection with more conventional CF pathogens, allergic bronchopulmonary aspergillosis (ABPA), and inadequate airway clearance.

Finally, to meet the microbiological criteria, patients should have two or more positive sputum cultures of the same NTM species or one positive culture from bronchoscopic lavage or wash [24].

### 7.2. Treatment

The decision to treat a patient who meets ATS/IDSA criteria for NTM-PD is not obvious and may not overlook factors such as NTM species involved, drug-related toxicity, adherence to the therapy, and expected outcomes of treatment; in addition, in paediatric patients, it is mandatory to minimize long-term drug-related toxicity and exposure to broad-spectrum antibiotics with consequent selective pressure for multiresistant bacteria [23,24].

The antibiotic regimen, which includes multiple drugs and whose duration usually reaches approximately 12–18 months, depends on the NTM species identified and consequently on its phenotypic and genotypic drug sensitivity; however, in patients with CF, the real effectiveness of different drugs is limited [21].

Macrolides are a standard tool in the treatment of NTM-PD, which explains the growing concern about emerging macrolide-resistant NTM species. The erythromycin ribosomal methylase (*erm*) gene, which confers inducible resistance to these drugs, has been detected in many RGMs, including the *erm* (41) gene in MAbsC [25].

Among the three subspecies of MAbsC, *M. abscessus* and *M. bolletii* exhibit extensive intrinsic and acquired resistance mechanisms due to a full-length functional *erm* (41) gene, while *M. massiliense* exhibits reduced resistance because of partial *erm* (41) gene expression [23]. Although consensus guidelines suggest preferential use of azithromycin over clarithromycin to limit inducible macrolide resistance, not all authors agree that the latter is a stronger inducer of the *erm* gene (41) than azithromycin [23,24].

Another study instead showed that *M. a. abscessus* may be less sensitive to clarithromycin than azithromycin, in contrast to *M. a. massiliense*, which appeared sensitive to both macrolides [21].

The resistance of *M. a. abscessus* to macrolides, streptogramins, and lincosamides may be mediated by mutations in the 23S rRNA.

The most active drugs for the treatment of MAbsC infection, in addition to azithromycin, are amikacin (IV, nebulized), cefoxitin (IV), and linezolid (IV, PO) [23,24]. Treatment should theoretically be continued 12 months beyond the last negative sputum culture in accordance with consensus guidelines, but it is burdensome, and in up to 50% of patients with MAC infection and in almost 100% of patients with MAbsC, it is interrupted because of its duration and adverse effects.

In CF patients with MAbsC infection, the treatment leads to improvement in FEV1 that is not equal between different time points compared to baseline (Table 7). As reviewed from DaCosta et al., the lower the starting lung function, the greater the improvement after therapy [21]. No data on other paediatric chronic respiratory diseases are available. 

## 8. NTM Cutaneous Infections

The incidence of cutaneous NTM infection is rare in children: they are more commonly seen in immunocompetent children but must also be considered in chronically immunosuppressed patients. [26] Common NTMs causing cutaneous disease include both rapidly growing mycobacteria (e.g., *Mycobacterium fortuitum*, MAbsC, *Mycobacterium chelonae*) and slow-growing mycobacteria (e.g., *Mycobacterium marinum*, *Mycobacterium ulcerans*) [27].

MAbsC causes the majority of skin infections, while other studies report the role of this pathogen in soft tissue infections [28]. The skin lesions of cutaneous NTM infection may present as a sporotrichoid pattern or abscesses or nodules, usually in a single-site infection.

MAbsC, *M. fortuitum,* and *M. chelonae* infections commonly occur after puncture or open wounds that come into contact with contaminated water and typically affect the lower extremities, while *M. marinum* infections occur from exposure of traumatized skin to contaminated water from aquariums or swimming pools. Disseminated systemic disease is rare in immunocompetent children, who usually present with isolated disease, but cutaneous NTM infection can be an early sign of disseminated systemic disease in an immunocompromised host (*M. marinum*, *M. ulcerans*, and *M. haemophilum* are the most common cause of cutaneous NTM infection associated with disseminated disease).

### Treatment

The treatment for localized uncomplicated cutaneous NTM would include a macrolide (azithromycin, clarithromycin) associated with a quinolone, tetracycline (doxycycline), or trimethoprim. In case of abscess, surgical drainage must be performed.

The general recommended duration of antibiotics in immunocompetent children with isolated cutaneous lesions includes a further 1 to 2 months after the resolution of symptoms, with a total duration of at least 3 to 4 months. Intravenous therapy, such as with amikacin and a carbapenem (e.g., meropenem, imipenem), may be required, in addition to a macrolide, for more extensive cutaneous disease complicated by ulceration, deep soft tissue infection, or osteomyelitis.

## 9. Disseminated NTM Infections

The incidence of disseminated NTM infections is approximately 0.2 cases/100,000 population, and they are mainly caused by MAC or *Mycobacterium kansasii* in HIV-infected patients with CD4+ count less than 50 cells/μL, with the same prevalence among adults and children, or in other severely immunocompromised people with transplants or haematologic malignancies [29,30].

To diagnose disseminated NTM infection, one or more isolates from blood, cerebrospinal fluid, bone marrow, pericardial, or peritoneal fluid is needed. They often show severe drug resistance in HIV-positive patients: a Chinese study demonstrated 100% isoniazid resistance, and 94.9% rifampicin and streptomycin resistance, with a relatively lower number of unresponsive infections treated with ethambutol (52.5%) and clarithromycin (33.9%) [31].

Moreover, only a few cases (one of these in a 1-year-old child) of disseminated *Mycobacterium chimaera* infections are described in patients who underwent open heart surgery with implantation of prosthetic material; these conditions can present with symptoms of cardiac or systemic involvement even after years, with poor outcomes.

ATS/IDSA guidelines advise a minimum of 12 months of therapy continuation for HIV patients with disseminated NTM infections after being asymptomatic and with CD4+ count greater than 100 cells/μL [32]. In particular, both HIV infection, to improve the patient’s immune system, and NTM infection should be treated at the same time to avoid adverse drug effects and drug–drug interactions. The treatment regimen should include a macrolide (clarithromycin 1000 mg/day or 500 mg twice daily rather than azithromycin 500 mg daily) and ethambutol 15 mg/kg. Rifampicin (or rifabutin 300 mg daily) can be added. If the susceptibility test shows macrolide resistance, amikacin or moxifloxacin should be administered. Moreover, azithromycin (1200 mg/week) is recommended as a preventive therapy in all HIV-infected patients with fewer than 50 CD4^+^ T cells/µL [33].

## 10. Conclusions

Recent literature points out that NTMs are ubiquitous, with several species including both those that are pathogens for humans and those that are not. This means that, if a mycobacterium is isolated from a patient’s specimen, we have to distinguish between a simple colonization and an NTM-related disease. The start of treatment start depends on many factors that are necessary to consider, such as clinical and imaging features, patient comorbidity and immunocompetence, drug adverse effects, and compliance with a very long therapy that can last many months.

Worldwide studies consistently document that, in contrast with adults, in whom pulmonary involvement is more frequent, immunocompetent children aged from 1 to 5 years show almost exclusively NTM-related lymphadenopathy. In these cases, surgical treatment with complete excision is considered the core treatment whenever possible because of its low rate of functional and cosmetic complications. Antibiotic therapy with multiple agents should be considered in cases of incomplete excision, fluctuant lesions, or when surgical treatment cannot be provided. Moreover, according to recent studies, the “wait-and-see” strategy, always associated with a strict follow-up, is an option for firm lesions. 

On the other hand, children with local risk factors, such as cystic fibrosis, may present NTM-pulmonary disease with a significant impact on respiratory functionality tests. The management of these infections is often burdensome for patients and for health providers. In these cases, long-lasting multiple antibiotic therapy is associated with many adverse drug effects that sometimes lead to treatment interruption. As a further complication, the high rates of resistant strains, particularly macrolide agents, must be taken into consideration.

Last, as described in adult patients, disseminated non-tuberculous mycobacterial infections usually occur only in severely immunocompromised individuals, in particular HIV-infected patients with CD4+ counts lower than 50 cells/µL. An association of two or more antibiotics, chosen according to mycobacteria sensitivity tests, is the recommended therapy. When treating these patients, drug-to-drug interactions should be taken into account to provide proper therapy for both the underlying condition and NTM disease.

Due to the clinical relevance of NTMs, whose prevalence is also increasing in children, guidelines for their optimal management, especially in the presence of chronic underlying disease, are urgently needed.

## Figures and Tables

**Table 1:** Most clinically relevant slow- and rapid-growing non-tuberculous mycobacteria (NTMs).

SGMs	RGMs
***M. avium***	*M. abscessus* ssp. *abscessus*
***M. intracellulare***	*M. abscessus* ssp. *massiliense*
***M. kansasii***	*M. abscessus* ssp. *bolletii*
***M. gordonae***	*M. immunogenum*
***M. lentiflavum***	*M. chelonae*
***M. malmoense***	*M. fortuitum*
***M. ulcerans***	*M. peregrinum*
***M. xenopi***	*M. septicum*
***M. marinum***	*M. mucogenicum*

SGMs, slow-growing mycobacteria; RGMs, rapid-growing mycobacteria.

**Table 2:** Diseases caused by NTMs and frequently isolated species.

Disease	Main Pathogens
Pulmonary infection	*M. abscessus*, *M. avium complex*, *M. kansasii*, *M. xenopi*, *M. malmoense*
Skin and soft tissue infections	*M. marinum*, *M. ulcerans*, *M. chelonae*, *M. abscessus*
Eye infections	*M. chelonae*, *M. abscessus*, *M. fortuitum*
Skeletal infections (osteomyelitis)	*M. abscessus*
Otitis media (middle ear infection), mastoiditis	*M. abscessus*, *M. kansasii*, *M. xenopi*
Cervical lymphadenitis (mainly in children)	*M. avium complex*, *M. malmoense*, *M. scrofulaceum*, *M. haemophilum*
Catheter- or device-associated infections	*M. abscessus*, *M. chelonae*
Cystic fibrosis (pulmonary infections)	*M. abscessus complex*, *M. avium complex*, *M, chelonae*
Disseminated disease (with immunosuppression or genetic predisposition)	*M. avium complex*
Post transplantation	*M. avium complex*, *M. kansasii*

**Table 3:** Risk factors for NTM infection.

Systemic Risk Factors	Local Risk Factors
SCID, CGD	Skin barrier disruption (laser cosmetic surgery, catheter)
Haematological malignancies	Corneal integrity alteration (LASIK)
GVHD	Lung disease: cystic fibrosis, tuberculosis, bronchiectasis itself, COPD, asthma, air pollution
HIV with CD4+ cells T count below 50/μL	
Iatrogenic immunosuppression (steroids, immunosuppressive and biological drugs)	
Genetic polymorphism or mutations in IL12/IFN-gamma pathways (MSMD)	
Vitamin D deficiency or vitamin D receptor polymorphisms	

SCID: severe combined immunodeficiency; CGD: chronic granulomatous disease, GVHD: graft-versus-host disease; COPD: chronic obstructive pulmonary disease.

**Table 4:** Clinical features of non-tuberculous mycobacterial lymphadenitis in children.

Clinical Features of NTM Lymphadenitis in Children
Unilateral lymph node in the jaw angle
Mobile lymph node
Hyperaemic skin
Possible fistula
Soft consistency on palpation

**Table 5:** Recommended antibiotic therapy for NTM lymphadenitis in children.

Antibiotic Therapy for NTM Lymphadenitis in Children
Clarithromycin 15–30 mg/kg/day in two doses or azithromycin 10–12 mg/kg/day
Rifampicin 350 mg/m^2^ body surface
Ethambutol 850 mg/m^2^ body surface

**Table 6:** American Thoracic Society and Infectious Diseases Society of America (ATS/IDSA) clinical and microbiological criteria for the diagnosis of pulmonary disease due to NTMs.

Clinical Criteria (Both Required)
1. Pulmonary symptoms, nodular or cavitary opacities on chest radiograph, or a high-resolution computed tomography scan that shows multifocal bronchiectasis with multiple small nodules.
2. Appropriate exclusion of other diagnoses.
Microbiologic Criteria (One of The Following Required):
1. Positive culture results from at least two separate expectorated sputum samples.
2. Positive culture result from at least one bronchial wash or lavage.
3. Transbronchial or other lung biopsy with mycobacterial histopathologic features (granulomatous inflammationor Acid-Fast Bacillus positive test) and positive culture for NTMs or biopsy showing mycobacterial histopathologic features (granulomatous inflammation or AFB) and one or more sputum or bronchial washings that are culture positive for NTMs.

**Table 7:** Antibiotic combinations for the treatment of non-tuberculous mycobacteria- pulmonary disease (NTM-PD) due to *Mycobacterium avium* complex (MAC) and *Mycobacterium abscessus* complex (MAbsC) [21].

*Mycobacterium abscessus* Complex	Macrolide Sensitive	Macrolide Resistant
**Non-severe disease** **Continue treatment for a minimum of 12 months after culture conversion**	Rifampicin Ethambutol Azithromycin or clarithromycin	Rifampicin Ethambutol Isoniazid Moxifloxacin Consider IV or nebulized amikacin for 2–3 months
**Severe disease** **Continue treatment for a minimum of 12 months after culture conversion**	Rifampicin Ethambutol Azithromycin or clarithromycin Consider IV or nebulized amikacin for 2–3 months	Rifampicin and Ethambutol with Isoniazid and/or moxifloxacin Consider amikacin IV for 2–3 months then nebulized for an additional 2–10 month period
**Initiation phase** **Aim for 4–5 active drugs** **Continue for 6–12 weekscourse**	First-line treatment Azithromycin or clarithromycin PO Amikacin IV Tigecycline, imipenem/meropenem Cefoxitin IV Clofazimine PO	
**Additional antibiotics to consider**	Bedaquiline PO (can be used for up to 6 months if available) If susceptible: Linezolid PO and/or moxifloxacin PO and/or cotrimoxazole PO and/or minocycline/doxycycline PO	
**Continuation phase** **Aim for 3 active drugs** **Continue 12 months after culture conversion**	Azithromycin or clarithromycin PO Amikacin nebulized Clofazimine PO	Amikacin nebulized Clofazimine PO (Azithromycin or clarithromycin PO) 1–2 of the following Linezolid PO Minocycline/doxycycline PO Moxifloxacin PO Cotrimoxazole PO

In bold, clinical findings and phase of treatment.
